# I THINK I KNOW HOW YOU FEEL: NEUROPSYCHOLOGICAL CORRELATES OF EMOTION PERCEPTION IN FRONTOTEMPORAL DEMENTIA

**DOI:** 10.1093/geroni/igae098.4212

**Published:** 2024-12-31

**Authors:** Anna Toledo, Nishita Paruchuri, Sae Yokoyama, Enna Chen, Alice Hua, Joel Kramer, Robert Levenson, Casey Brown

**Affiliations:** Georgetown University, Washington, District of Columbia, United States; Georgetown University, Washington, District of Columbia, United States; Princeton University, Princeton, New Jersey, United States; Stanford University, Stanford, California, United States; UC Berkeley, UC Berkeley, California, United States; University of California, San Francisco, University of California, San Francisco, California, United States; University of California, Berkeley, Berkeley, California, United States; Georgetown University, Georgetown University, District of Columbia, United States

## Abstract

Neurodegenerative diseases lead to deficits in cognitive functioning that could disrupt the ability to perceive others’ emotions. We examined whether cognitive deficits in semantic knowledge and executive function relate to two aspects of emotion perception. Individuals with frontotemporal dementia and healthy controls (N = 110; 33 behavioral variant, 23 non-fluent variant, 30 semantic variant, and 24 healthy controls) completed cognitive tests of semantic word knowledge (Peabody Picture Vocabulary Test and Boston Naming Test) and executive function (Digit Span Backwards, Stroop, Design Fluency, and Trail-Making). To assess different facets of emotion perception, participants completed an emotion labeling task that measured the ability to identify specific emotions (sad, happy) experienced by characters in films and an emotion tracking task that measured the ability to continuously track the emotional valence of a character in a film using a rating dial. Regression analyses revealed that semantic word knowledge was associated with emotion labeling but not emotion tracking, even after accounting for relevant covariates (diagnosis, age, sex, global cognition, and dementia severity). Executive function was associated with both emotion labeling and emotion tracking, even when accounting for covariates. Steiger’s Z test revealed that executive function was more strongly associated with emotion valence tracking than with emotion category labeling. Findings reveal how distinct neurocognitive abilities are linked with unique facets of emotion perception. Semantic word knowledge may subserve the ability to distinguish between specific emotion categories and executive function may be more important when continuously updating one’s unfolding emotions than making a single emotional judgment.

